# Influence of Single Layer Centrifugation with Canicoll on Semen Freezability in Dogs

**DOI:** 10.3390/ani12060714

**Published:** 2022-03-11

**Authors:** Guillaume Domain, Hiba Ali Hassan, Eline Wydooghe, Osvaldo Bogado Pascottini, Anders Johannisson, Jane M. Morrell, Wojciech Niżański, Ann Van Soom

**Affiliations:** 1 Department of Internal Medicine, Reproduction and Population Medicine, Faculty of Veterinary Medicine, Ghent University, Salisburylaan 133, 9820 Merelbeke, Belgium; hiba.alihassan@ugent.be (H.A.H.); eline.wydooghe@vives.be (E.W.); osvaldo.bogado@ugent.be (O.B.P.); ann.vansoom@ugent.be (A.V.S.); 2 Veterinary Physiology and Biochemistry, Department of Veterinary Sciences, University of Antwerp, 2610 Wilrijk, Belgium; 3 Department of Clinical Sciences, Division of Reproduction, Swedish University of Agricultural Sciences, 756-51 Uppsala, Sweden; anders.johannisson@slu.se (A.J.); jane.morrell@slu.se (J.M.M.); 4 Department of Reproduction and Clinic of Farm Animals, University of Environmental Science, Grundwaldzki Square 49, 50-357 Wroclaw, Poland; wojciech.nizanski@upwr.edu.pl

**Keywords:** canine, fertility, semen selection, cryopreservation, semen quality

## Abstract

**Simple Summary:**

Freezing dog semen is not always possible due to low quality sperm or poor survival during freezing. In order to make this assisted reproductive technique available to a larger number of dogs, this study investigated the benefit of selecting the best spermatozoa before freezing using single layer centrifugation (SLC). The results indicated that this technique was effective in separating spermatozoa according to their quality, although this resulted in losing some good quality spermatozoa. After thawing, spermatozoa centrifuged by SLC were of better quality than after standard centrifugation. However, spermatozoa from suboptimal quality semen did not survive freezing as well as spermatozoa from semen of optimal quality, even after SLC. Single layer centrifugation, therefore, makes it possible to obtain better quality spermatozoa after thawing but is not sufficient on its own to improve the inferior freezing ability of spermatozoa from suboptimal quality semen. So far, eighteen pups were born after insemination with SLC-selected frozen-thawed semen, proving that these selected spermatozoa remain fertile.

**Abstract:**

This study evaluated how semen selection by single layer centrifugation (SLC) with Canicoll affects semen freezability in dogs. A total of eighteen ejaculates, collected from dogs with optimal and suboptimal semen quality (optimal: normal morphology (NM) ≥ 80%, *n* = 9; suboptimal: NM between 60 and 79%, *n* = 9), were divided into two aliquots and subjected to standard centrifugation or SLC before cryopreservation. Motility, NM, membrane integrity, mitochondrial membrane potential (MMP), and DNA integrity were improved in fresh samples after SLC, regardless of semen quality, but at the expense of some good quality spermatozoa. After thawing, NM and membrane integrity were improved in SLC-selected semen in both semen qualities. Interestingly, MMP was also higher but only in optimal quality semen. Still, spermatozoa from suboptimal quality semen did not survive freezing to the same extent as spermatozoa from optimal quality semen, even after selecting superior spermatozoa. Semen selection with Canicoll is, therefore, an effective technique to isolate a subpopulation of high-quality spermatozoa and obtain sperm samples of better quality after thawing, but is not sufficient to improve the intrinsic inferior freezability of suboptimal quality semen. So far, eighteen pups were born after insemination with SLC-selected frozen-thawed semen, proving that these selected spermatozoa remain fertile.

## 1. Introduction

In dogs, cryopreservation of semen has become a well-known assisted reproduction technique for breeders. This technique allows the genetics of superior dogs to be preserved and exchanged with no restriction in time or distance [1,2,3]. In comparison to other species, the plasma membrane of dog spermatozoa contains a high cholesterol-to-phospholipids ratio, which makes themrelatively resistant to cold and osmotic stress [4]. Nevertheless, cryopreservation may cause major damage to canine spermatozoa and unfortunately, the quality of thawed semen is highly variable among dogs and ejaculates [5,6]. This variability has also been observed in other species and has led to the ranking of stallions and bulls according to the freezability of their ejaculates [7,8]. The use of so-called “good freezers” in these species has optimized their breeding efficiency but this method is not yet applicable in dogs, since a large number of dog breeds are only represented by a small and closed population. Hence, further restriction on the use of stud dogs based on semen freezability would shrink the genetic pool even more, leading to more inbreeding [9,10,11,12]. Therefore, efforts should be made to improve semen cryopreservation from samples with suboptimal quality and/or samples with poor semen freezability. In this respect, the selection of a subpopulation of superior spermatozoa could possibly be an approach to allow for better survival during cryopreservation. This hypothesis is based on the fact that the presence of more senescent, damaged, non-motile, immature and/or dead spermatozoa in lower quality ejaculates might be deleterious to the survival of good quality spermatozoa, partly due to the production of reactive oxygen species (ROS) [13].

Reactive oxygen species cause damage to many cellular structures, including spermatozoa. Sperm cells are extremely sensitive to ROS because of the high content of polyunsaturated fatty acids in their plasma membrane and their limited antioxidant systems [14,15]. When an imbalance between ROS generation and ROS neutralization occurs in the semen, oxidative stress ensues and induces lipid peroxidation, loss of plasma membrane integrity, axonemal damage, structural DNA damage and apoptosis of the spermatozoa [16]. To limit such damage, several methods are available to select functional spermatozoa from nonfunctional, abnormal spermatozoa (i.e., migration, filtration, and colloid centrifugation) [17]. Amongst these techniques, colloid centrifugation appears to be the most efficient by selecting spermatozoa based on a variety of semen characteristics such as motility, morphology, plasma membrane integrity, acrosome integrity, resistance to lipid peroxidation, and unfragmented DNA [17]. Bacterial and viral contaminations are also highly reduced after selection [18,19,20,21,22]. Single layer centrifugation (SLC) consists of centrifugation through only one layer of colloid. It is simpler to use than density gradient centrifugation, while being equally efficient [17]. In dogs, SLC was shown to select spermatozoa based on motility [23,24], morphology [24,25], viability [24], acrosome integrity [24], and DNA integrity [26] from chilled and frozen-thawed semen. However, no study has investigated the effect of SLC on canine semen freezability although its value has already been demonstrated in stallion [20,21,27], boar [28,29], and bull spermatozoa [30].

The objective of this study was to evaluate the effect of SLC on semen freezability in dogs. Optimal and suboptimal ejaculates were investigated separately to determine whether the effect of SLC was influenced by the initial semen quality. The hypothesis was that SLC would have a greater positive effect on ejaculates of suboptimal quality, which usually do not freeze well.

## 2. Materials and Methods

### 2.1. Animals

A total of twenty-four intact, privately-owned male dogs were enrolled in the present study at the teaching hospital of Ghent University between July 2020 and February 2021. All dogs were at least 1 year old, of medium to large breeds, clinically healthy, and had not been sick or given medication during the last 6 months. Exclusion criteria were abnormal clinical examination, bad semen quality (normal morphology (NM) < 60%), and a total sperm output (TSO) lower than 400 × 10^6^ spermatozoa. This last exclusion criterion was chosen to ensure sufficient spermatozoa were available for freezing after SLC. Six dogs were excluded from the initial pool due to oligozoospermia, bad semen quality, or benign prostate hyperplasia. The final population consisted of 18 dogs from 13 breeds with a mean age of 38 months.

Two groups of dogs were investigated based on semen quality (optimal, *n* = 9; suboptimal, *n* = 9). An ejaculate was considered of optimal quality when NM ≥ 80% [31,32] and suboptimal when NM was between 60 and 79%. The threshold value of 60% was chosen since an ejaculate containing more than 40% of morphologically abnormal spermatozoa should be classified as poor quality as it may reflect disturbances in testicular and/or epididymal function [31,32]. Below this threshold, a dog’s fertility is drastically affected and semen cryopreservation should be discouraged [33].

### 2.2. Semen Collection and Processing

**Figure 1:**
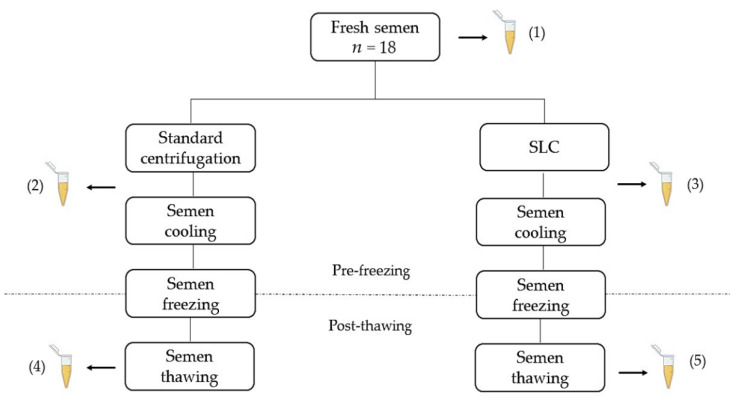
Flow chart describing the experimental design. Treatment groups: (1) = fresh semen; (2) = standard centrifuged semen; (3) = single layer centrifugates (SLC)-selected semen; (4) = standard centrifuged semen post-thawing; (5) = SLC-selected semen post-thawing. *n* = 18 semen samples (9 of optimal (normal morphology ≥ 80%) and 9 of suboptimal (normal morphology between 60 and 79%) semen quality).

The sperm-rich fraction of each ejaculate was collected by digital manipulation into plastic vials as described by Linde-Forsberg [1]. After collection, the semen was placed in an incubator at 37 °C and immediately assessed by a single operator (as described below). If the semen quality met the selection criteria (optimal or suboptimal quality with a TSO ≥ 400 × 10^6^ spermatozoa), the semen was diluted to a concentration of 100 × 10^6^/mL in a warm TRIS-citric acid-glucose-based extender containing 20% of egg yolk [34] and further processed. The dilution of the raw semen to a concentration of 100 × 10^6^/mL has been found to optimize the yield of colloid centrifugation in stallions [35] and dogs (unpublished data). After dilution, semen was divided into two aliquots. The first aliquot was centrifuged at 720× *g* for 5 min at 22 °C (standard centrifugation) [34]. The second aliquot was subjected to SLC following a modification of the procedure described by Dorado et al. [25]. In brief, 2 mL of extended semen was layered on top of 2 mL of colloid (Canicoll; Swedish University of Agricultural Sciences—SLU, Uppsala, Sweden), previously equilibrated to room temperature (22 °C), and the preparation was centrifuged at 300× *g* for 20 min at 22 °C [35]. Supernatants were discarded, together with the colloid for the SLC-processed samples, and pellets were diluted to a concentration of 200 × 10^6^/mL in a freezing extender containing 3% glycerol (Uppsala I [36]). Extended semen was equilibrated at 4 °C for 90 min after an aliquot was set aside for analysis (as described below). After equilibration, semen was diluted to a concentration of 100 × 10^6^/mL in the second freezing extender containing 7% glycerol (Uppsala II [36]) and immediately frozen in 0.5 mL straws in a computerized freezing machine (IceCube 1810; SyLab, Purkersdorf, Austria) following the freezing curve described by Schafer-Somi et al. [37]. At the end of the program, straws were plunged into liquid nitrogen and stored for at least one week. One straw per treatment was then thawed in a 37 °C water bath for 30 s and semen quality was evaluated after 5 min incubation at 37 °C (as described below). In total, 5 samples per ejaculate were examined at different steps of the protocol (Figure 1).

### 2.3. Semen Quality Assessment

#### 2.3.1. Concentration

Semen concentration was measured using the Nucleocounter-SP100 (ChemoMetec, A/S, Allerød, Denmark), according to the manufacturer’s instructions [38]. Briefly, a 10 µL aliquot of semen was diluted 101 times with 1 mL of lysis reagent S100 (ChemoMetec, A/S, Allerød, Denmark) and loaded into a SP1 cassette (ChemoMetec, A/S, Allerød, Denmark) containing propidium iodide (PI). The cassette was then inserted into the fluorescence detector of the machine and the semen concentration of the sample was reported. Total sperm output was obtained by multiplying concentration by semen volume.

#### 2.3.2. Motility and Velocity

The computer-assisted sperm analyzer ISAS^®^v1 (Proiser, Valencia, Spain) equipped with a heated stage set at 37 °C and a 10× negative phase-contrast objective was used to measure motility and velocity parameters. A total of thirty consecutive, digitized images were captured at a frame rate of 60 fps by the video digital camera (Proiser 782C, Proiser R + D, Paterna, Spain) of the analyzer. A spermatozoon was considered immotile when presenting a VAP < 10 µm/s and spermatozoa which deviated <50% from a straight line were designated as progressive. Tail detection was activated to allow non-sperm particles to be ignored and particle area was set between 12 and 80 µm^2^. The playback function was used to minimize the number of objects incorrectly identified as spermatozoa.

Before analysis, each sample was diluted to a working concentration of 40 × 10^6^ cells/mL in physiological saline solution [39,40]. A 4 µL droplet was then loaded in a pre-warmed ISAS^®^D4C20 disposable counting chamber (Proiser, Valencia, Spain) and five fields were captured and analyzed. For each field, ten kinematic parameters were reported and the average of the five fields was taken for analyses: total motility (TM, %), PM (%), average path velocity (VAP, µm/s), straight line velocity (VSL, µm/s), curvilinear velocity (VCL, µm/s), amplitude of lateral head displacement (ALH, µm), beat cross frequency (BCF, Hz), wobble (WOB, %), straightness (STR, %) and linearity (LIN, %).

#### 2.3.3. Morphology

Morphology of spermatozoa was assessed on eosin/nigrosin stained smears under bright-field microscopy at 1000× magnification (Olympus BX51TF, Tokyo, Japan). One hundred spermatozoa per sample were evaluated and classified according to their morphology: normal (NM), abnormal head (AH), abnormal midpiece/tail (AMT), proximal protoplasmic droplet (PPD), and distal protoplasmic droplet (DPD) [34].

#### 2.3.4. Plasma Membrane Integrity, Acrosome Integrity and Mitochondrial Membrane Potential

These characteristics were analyzed simultaneously using a modification of the triple fluorescent procedure described by Angrimani et al. [41], which includes the fluorochromes PI, fluorescein isothiocyanate peanut (*Arachis hypogaea*) agglutinin (FITC-PNA), and 5,5′,6,6′-tetrachloro-1,1′,3,3′-tetraethylbenzimidazolylcarbocyanine iodide (JC-1). All reagents were purchased from Sigma-Aldrich (Bornem, Belgium) and the detailed composition of HEPES-TALP can be found in the study by Rijsselaere et al. [34].

Briefly, an aliquot of semen was diluted in HEPES-TALP medium to a concentration of 25 × 10^6^ cells/mL and 150 µL of the diluted sample was placed into a microcentrifuge tube. Then, 3 µL of 0.75 mM PI, 8 µL of 0.1 mg/mL FITC-PNA, and 8 µL of 153 µM JC-1 were added and the sample was incubated for 8 min at 37 °C in the dark. After incubation, a 10 µL aliquot was mounted on a pre-warmed slide, covered with a coverslip, and evaluated using a Leica DMR epifluorescence microscope (Leica Microsystems, Wetzlar, Germany) equipped with a mercury lamp at a magnification of 1000×. One hundred spermatozoa per sample were counted and classified into eight different classes according to their staining patterns (Table 1) [41]. Intact plasma membrane was obtained by adding classes I to IV, acrosome integrity by adding classes I-II-V-VI, and high MMP by adding classes I-III-V-VII.

#### 2.3.5. Oxidative Stress

Semen lipid peroxidation levels were evaluated by measuring the concentration of thiobarbituric acid (TBA) reactive substances (RS) generated after challenging spermatozoa to an ROS generating system [42]. During this challenge, malondialdehyde (MDA) is produced as one of the final products of polyunsaturated fatty acids peroxidation in the cells [43] and after incubation with TBA at high temperature and low pH, two molecules of TBA and one molecule of MDA will react and result in TBA-RS, a pink color complex that can be quantified with a spectrophotometer.

In brief, semen was diluted to a concentration of 100 × 10^6^ cells/mL in Dulbecco’s phosphate-buffered saline (DPBS) and 200 µL of the diluted semen was incubated with 50 µL of 4 mM ferrous sulfate and 50 µL of 20 mM sodium ascorbate at 37 °C for 90 min in the dark. If extended, semen was first washed twice with DPBS to remove the diluent. Subsequently, 600 µL of 10% trichloroacetic acid (v:v) at 4 °C was added and the sample was centrifuged at 21.130× *g* at 4 °C for 15 min to promote the precipitation of proteins and debris. After centrifugation, 500 µL of the supernatant was recovered and mixed with 500 µL of 1% TBA (v:v). The sample was incubated at 100 °C for 15 min after which it was immediately cooled in an ice bath to stop the chemical reaction. Finally, the TBA-RS concentration was quantified with a spectrophotometer (Multiskan GO, Thermo Fisher Scientific, Waltham, MA, USA) at a wavelength of 532 nm and expressed as nanograms of TBA-RS/10^6^ spermatozoa. This assay was performed three times for each sample and the mean was taken as the result.

#### 2.3.6. Sperm Chromatin Structure Assay (SCSA)

Sperm chromatin integrity was assessed with acridine orange (AO) based on the methodology reported by Evenson et al. [44]. Briefly, semen was diluted to a concentration of 10 × 10^6^ cells/mL in DPBS. An aliquot of 250 µL was then snap frozen and kept at −80 °C until further analysis. On the day of analysis, samples were thawed on crushed ice and diluted with TRIS-sodium chloride-EDTA (TNE) buffer (0.15 M NaCl, 0.01 M Tris-HCl, 1 mM EDTA, pH 7.4) to a concentration of 2 × 10^6^ cells/mL. Subsequently, 100 µL of the solution was subjected to partial DNA denaturation by the addition of 200 µL of an acidic detergent solution (0.17% Triton X-100, 0.15 M NaCl and 0.08 N HCl, pH 1.2). Thirty seconds later, 600 µL of AO (6 μg/mL in 0.1 M citric acid, 0.2 M Na_2_HPO_4_, 1 mM EDTA, 0.15 M NaCl, pH 6.0) was added and the stained samples were analyzed within 3–5 min using a BD LSR flow cytometer (Becton Dickinson, San Jose, CA, USA) equipped with standard optics. The fluorophore was excited with an argon ion laser at 488 nm and data collection was carried out using Cell Quest (version 3.3, Becton Dickinson). A total of 10,000 events were collected from each sample and the DNA fragmentation index (DFI, %) was calculated as the ratio of cells presenting denaturated, single-stranded DNA to the total number of cells acquired.

### 2.4. Recovery Rates

The recovery rates (RR) of different semen parameters (total sperm count (TSC), TM, PM, and NM) were calculated based on the values obtained after both centrifugations according to the following formula [25]:

$$RR\;\left(\%\right)=\frac{\begin{array}{c} Total\;sperm\;count\;after\;centrifugation\;\\ \left(x\;value\;of\;parameter\;after\;centrifugation\right) \end{array}}{\begin{array}{c} Total\;sperm\;count\;before\;centrifugation\\ \left(x\;value\;of\;parameter\;before\;centrifugation\right) \end{array}}\;\times \;100\;$$

### 2.5. Statistical Analysis

Statistical analyses were performed using R studio version 4.1.2 (R Inc., Boston, MA, USA). Variables were explored using histograms and the normality of the distributions was verified using the Shapiro—Wilk test. The effect of the centrifugation technique on semen parameters was fitted in linear mixed effect models using the lme4 and lmer Test packages [45]. Centrifugation techniques, semen quality and their interaction were forced into each model and dog was taken as a random factor. Arcsin transformations were used when the residuals of the model were not normally distributed (*p* < 0.05) [46]. The Tukey’s test was used to compare the differences between the estimates (cld function). The level of significance was set at *p* < 0.05 for all analyses and results are expressed as means and standard errors.

## 3. Results

### 3.1. Pre-Freezing

Standard centrifugation of optimal quality ejaculates decreased PM (*p* < 0.05), VCL (*p* < 0.01), BCF (*p* < 0.001), and TBA-RS (*p* < 0.001) while it increased WOB (*p* < 0.01) and AMT (*p* < 0.01) in comparison to fresh, unprocessed semen samples. Single layer centrifugation of these ejaculates, on the other hand, improved TM (*p* < 0.05), most velocity parameters (VSL, VAP, LIN, and WOB; *p* < 0.001), NM (*p* < 0.001), and high MMP (*p* < 0.01) and decreased TBA-RS (*p* < 0.001), BCF (*p* < 0.001), all abnormal morphologies (*p* < 0.05), and DNA fragmentation (*p* < 0.001) in comparison to unprocessed semen samples. Finally, SLC-selected spermatozoa from optimal quality ejaculates had an improved TM (*p* < 0.001), PM (*p* < 0.001), most velocity parameters (VCL, VSL, VAP, LIN, and WOB; *p* < 0.05), NM (*p* < 0.001), intact membrane integrity (*p* < 0.01), high MMP (*p* < 0.001), and decreased DNA fragmentation (*p* < 0.01) in comparison to standard centrifuged spermatozoa (Table 2).

Fresh ejaculates of suboptimal quality were similarly influenced by both centrifugation techniques. The differences with optimal quality ejaculates were that TM (*p* < 0.001) also decreased after standard centrifugation in comparison to fresh semen. Single layer centrifugation, on the other hand, also improved PM (*p* < 0.001) and STR (*p* < 0.001) in comparison to fresh semen. Finally, STR was also improved (*p* < 0.001) after SLC in comparison to standard centrifugation (Table 2). After SLC, the semen quality of suboptimal ejaculates was comparable to semen quality from optimal ejaculates.

#### Recovery Rates

**Figure 2:**
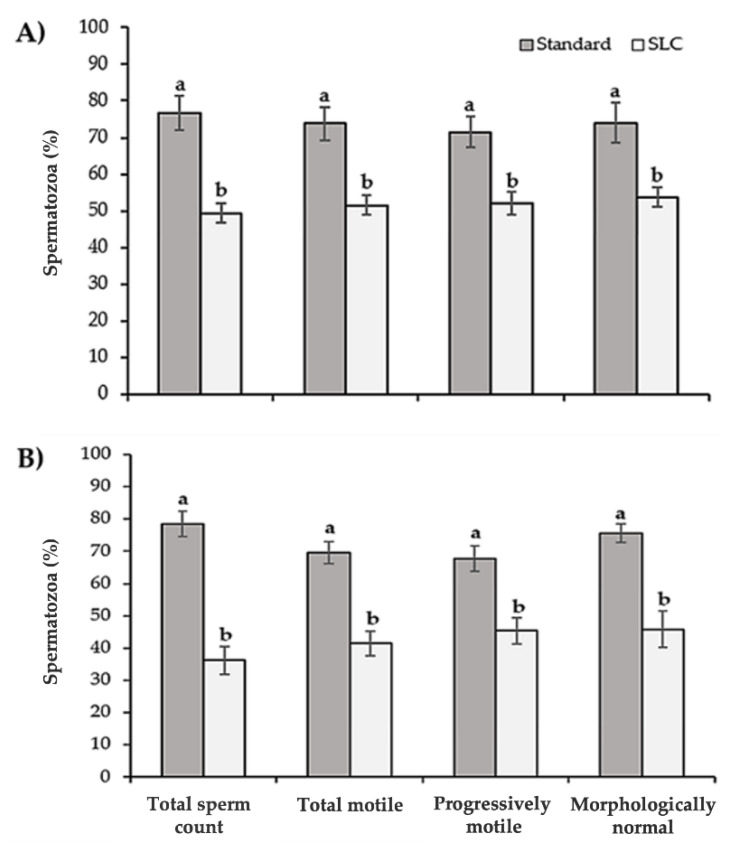
Recovery rates (means ± SEM) after standard centrifugation (in grey) or single layer centrifugation (SLC) (in white) of optimal (**A**, normal morphology ≥ 80%) and suboptimal (**B**, normal morphology between 60 and 79%) quality ejaculates. ^a^^,b^ Different superscripts indicate significant differences between treatments (*p* < 0.05).

With ejaculates of optimal quality, 76.7% of the initial TSO was recovered after standard centrifugation in comparison to 49.4% after SLC (Figure 2). A similar percentage (78.6%) was recovered with suboptimal quality ejaculates after standard centrifugation but only 36.2% of spermatozoa were recovered after SLC. In both semen qualities, the recovery rates of motile, progressive motile and morphologically normal spermatozoa were lower (*p* < 0.01) after SLC in comparison to standard centrifugation.

### 3.2. Post-Thawing

After thawing, SLC-selected semen from optimal quality ejaculates had an increased LIN (*p* < 0.01), WOB (*p* < 0.001), NM (*p* < 0.001), intact plasma membrane (*p* < 0.05), high MMP (*p* < 0.01) and a decreased VCL (*p* < 0.01) compared to standard centrifuged spermatozoa (Table 3). In addition, all abnormal morphologies were still decreased in SLC-selected semen (*p* < 0.05), except for AH.

Suboptimal quality ejaculates had the same improved parameters as optimal ejaculates after thawing, except for the high MMP, which was not different (*p* > 0.05) from that of the standard centrifuged semen (Table 3). Finally, SLC-selected spermatozoa from suboptimal quality ejaculates had a trend (*p* = 0.05) for a lower DNA fragmentation index in comparison to standard centrifuged spermatozoa.

Interestingly, SLC-selected spermatozoa from suboptimal quality ejaculates had a lower quality (*p* < 0.05) post-thawing than spermatozoa from optimal quality ejaculates. This was seen in particular at the level of motility with a difference of 20%, but also at the level of the plasma membrane and acrosome integrity with a difference of 10% between the two qualities.

## 4. Discussion

The present study provides evidence that SLC with Canicoll prior to freezing improves some aspects of semen quality after thawing. However, the cryosurvival of suboptimal quality ejaculates remains lower than that of optimal quality ejaculates, even after selection for the best spermatozoa.

During canine semen cryopreservation, seminal plasma is usually removed by centrifugation to optimize semen cryosurvival [37]. However, standard centrifugation and dilution of the semen in the freezing extender adversely affect sperm motility as was described in the study of Schäfer-Somi et al. [37]. The present study found similar results, and this was especially true for semen of suboptimal quality whose spermatozoa are probably less robust and more likely to be affected by the physical stress of centrifugation. More spermatozoa with AMT were also found after standard centrifugation, although the percentage of morphologically normal spermatozoa was not affected. Apart from the decrease in some kinematic parameters and the increase of AMT, other semen parameters were not affected by standard centrifugation. Colloid centrifugation with Canicoll, on the other hand, modified numerous semen parameters prior to freezing. Interestingly, this improvement did not depend upon the initial quality of the semen although it was, as expected, greater for ejaculates of suboptimal quality. The subpopulation of SLC-selected spermatozoa had improved TM, PM, velocity parameters, NM, plasma membrane integrity, high MMP, and less DNA fragmentation than standard centrifuged spermatozoa. Interestingly, all abnormal morphologies investigated in this study were decreased after SLC. This is consistent with the role of Canicoll in selecting a subpopulation of higher quality spermatozoa and supports results obtained in other studies [23,24,26]. However, SLC did not seem to select canine spermatozoa with an intact acrosome, similarly to what was described in other studies [23,25], although a loss of acrosomal integrity induced by the colloid has not been ruled out yet.

The removal of seminal plasma prior to freezing also affects the number of spermatozoa that will be frozen. In this study, 77 to 79% of the spermatozoa were recovered after standard centrifugation in comparison to 49% and 36% (optimal and suboptimal quality semen, respectively) after SLC. These recovery rates of TSC after SLC are in line with those described by Martinez et al. [28] in boars and Dorado et al. [25] in dogs. This significant loss of spermatozoa was expected given the role of the colloid in removing low quality spermatozoa. However, the recovery rates of motile, progressively motile, and morphologically normal spermatozoa after SLC were also significantly lower than after standard centrifugation. This means that although SLC isolated good quality spermatozoa from a lower quality pool, it did so at the expense of a proportion of good quality spermatozoa.

After thawing, NM, plasma membrane integrity, some velocity parameters, and the percentage of spermatozoa having a high MMP (for optimal quality ejaculates) were still significantly improved in SLC-selected semen in comparison to standard centrifuged semen in both qualities. The percentage of AH was, however, no longer different between the two treatments indicating that SLC-selected spermatozoa had relatively more head damage than unselected spermatozoa. Investigation of plasma membrane modifications induced by SLC would therefore be interesting to understand the increased sensitivity of selected spermatozoa to cryo-induced head damage. Finally, the DNA fragmentation index of suboptimal quality ejaculates was still decreased in SLC-selected in comparison to standard centrifuged spermatozoa, although this difference was not significant anymore. These results are partially in agreement with what has been demonstrated in other species [20,27,28,29,30]. However, some differences could be found with these previously published studies. In boars, motility [28,29] and resistance to oxidative stress [28] of SLC-selected spermatozoa were improved in comparison to standard centrifuged spermatozoa while motility, DNA integrity, and acrosome integrity were improved in stallions [27]. Another study in stallions, however, did not confirm the improved motility in SLC-selected spermatozoa after thawing [20]. These varying conclusions may reflect interspecies peculiarities or be the result of the amount of spermatozoa investigated along with the different techniques and machines used to process and analyze the semen.

In the present study, lipid peroxidation was evaluated instead of ROS levels because the measurement of ROS does not provide any information regarding the damage induced to spermatozoa, whereas lipid peroxidation levels directly reflect this damage [43]. It might be surprising to note that centrifugation significantly decreased the level of TBA-RS obtained after exposing the semen to oxidative stress. This difference can however be explained by the presence of some seminal plasma during the oxidative challenge in non-centrifuged ejaculates. Seminal plasma not only contains some MDA [47], but the lipid content also produces MDA during the oxidative challenge [43]. After centrifugation and washing of the semen, on the other hand, almost all of the MDA production comes from plasma membrane oxidation of the spermatozoa. The lack of difference between the two centrifugation techniques suggests that SLC does not isolate spermatozoa more resistant to lipid peroxidation, at least not in dogs.

The classification of ejaculates according to their quality was solely based upon morphology. However, TM, PM, plasma membrane integrity, and DNA integrity were also significantly different between the two semen qualities (Table 1 and Table 2, statistical analyses not shown). Interestingly, it was found that ejaculates of suboptimal quality contained about 20% of spermatozoa with fragmented DNA, twice as much as ejaculates of optimal quality. This percentage is quite striking given that it has been shown in many species, including humans, that DNA fragmentation index is strongly linked to fertility [48,49]. Although no studies have yet been conducted on dogs, a dog with suboptimal semen quality may then be at greater risk of subfertility. Suboptimal semen quality can be caused by the age of the dog as older dogs are known to produce lower quality semen and semen with lower cryotolerance [50,51]. However, this parameter could not have influenced the results of this study as both groups were composed of dogs of similar age (42.0 ± 15.6 months and 34.7 ± 18.8 months (mean ± standard deviation) for optimal and suboptimal semen quality, respectively). The breed effect was also avoided as both groups were composed of comparable breeds and the TSO, known as the only parameter influenced by the breed, was not considered in this study [51,52]. Finally, no seasonal effect was to be considered in this study since semen quality is not influenced by the season in dogs [51].

In dogs, frozen semen is currently solely used for intrauterine insemination and this technique requires a substantial number of sperm cells to achieve adequate pregnancy rates, about 100–150 × 10^6^ motile spermatozoa [53]. Although intra-oviductal insemination or in vitro oocyte fertilization would drastically reduce the number of spermatozoa required (2–4 × 10^6^ and 0.1 × 10^6^ spermatozoa, respectively), these techniques are invasive, more complex, and currently lead to disappointing results in dogs [54,55]. For this reason, the loss of 51 to 64% of spermatozoa after SLC is an important factor restricting its use in dogs given the limited number of spermatozoa obtained in one ejaculate. This downside may, however, become secondary if the selected spermatozoa are shown to have better in vivo efficiency (i.e., a higher percentage of pregnancies and/or a higher number of pups per litter). At the time of publication, four bitches were inseminated with frozen-thawed spermatozoa from SLC-processed semen. Three got pregnant and gave birth to three puppies (Great Dane), seven puppies (Rottweiler), and eight puppies (Rottweiler). Further research is therefore needed to address this question.

## 5. Conclusions

The present study provides evidence that SLC with Canicoll can select good quality spermatozoa and that this selection before semen cryopreservation results in better quality semen after thawing. The cryosurvival of spermatozoa from suboptimal quality semen remains, however, lower than that of optimal quality semen, indicating that SLC is not the only treatment that should be undertaken to improve the freezability of these samples. Finally, the significant loss of spermatozoa after SLC may limit its use in current cryopreservation protocols unless other assisted reproductive technologies such as intra-oviductal insemination or in vitro oocyte fertilization become available in dogs or unless SLC-selected spermatozoa are shown to provide better in vivo performances.

## Figures and Tables

**Table 1:** Sperm classification by simultaneous assessment of plasma membrane integrity (propidium iodide—PI), acrosome integrity (fluorescein isothiocyanate peanut *(Arachis hypogaea)* agglutinin—FITC/PNA) and mitochondrial membrane potential (JC-1) by triple stain fluorescent probes.

Sperm Cell Pattern	PI	FITC-PNA	JC-1
Intact plasma membrane, intact acrosome, high MMP (I)	−	−	Orange
Intact plasma membrane, intact acrosome, low MMP (II)	−	−	Green
Intact plasma membrane, damaged acrosome, high MMP (III)	−	+	Orange
Intact plasma membrane, damaged acrosome, low MMP (IV)	−	+	Green
Damaged plasma membrane, intact acrosome, high MMP (V)	+	−	Orange
Damaged plasma membrane, intact acrosome, low MMP (VI)	+	−	Green
Damaged plasma membrane, damaged acrosome, high MMP (VII)	+	+	Orange
Damaged plasma membrane, damaged acrosome, low MMP (VIII)	+	+	Green

PI negative (*−*) = unstained nucleus, PI positive (+) = red stained nucleus, FITC-PNA negative (*−*) = unstained acrosome, FITC-PNA positive (+) = green stained acrosome. MMP = mitochondrial membrane potential.

**Table 2:** Semen parameters (mean ± SEM) of optimal quality ejaculates (normal morphology ≥ 80%) and suboptimal quality ejaculates (normal morphology between 60 and 79%) after collection (Fresh), standard centrifugation (Standard), and single-layer centrifugation (SLC) before freezing.

	Optimal Quality			Suboptimal Quality		
**Parameter**	**Fresh**	**Standard**	**SLC**	**Fresh**	**Standard**	**SLC**
Total motility (%)	89.0 ± 1.1 ^b^	85.6 ± 1.3 ^b^	92.9 ± 0.7 ^a^	75.7 ± 3.8 ^y^	66.7 ± 4.0 ^z^	88.4 ± 1.9 ^x^
Progressive motility (%)	81.0 ± 1.4 ^a^	75.7 ± 1.6 ^b^	85.2 ± 0.6 ^a^	64.4 ± 3.9 ^y^	55.3 ± 4.1 ^z^	82.2 ± 2.0 ^x^
Curvilinear velocity (µm/s)	175.3 ± 7.1 ^a^	167.0 ± 3.9 ^b^	174.1 ± 3.9 ^a^	164.8 ± 7.8 ^x^	150.9 ± 4.9 ^y^	160.8 ± 5.7 ^x^
Straight line velocity (µm/s)	108.9 ± 1.2 ^b^	111.1 ± 3.8 ^b^	124.4 ± 2.9 ^a^	96.1 ± 6.0 ^y^	93.9 ± 4.5 ^y^	117.4 ± 3.4 ^x^
Average path velocity (µm/s)	131.7 ± 1.7 ^b^	133.7 ± 4.1 ^b^	147.8 ± 2.5 ^a^	118.3 ± 5.9 ^y^	114.3 ± 4.6 ^y^	135.9 ± 4.3 ^x^
Linearity (%)	62.4 ± 2.2 ^b^	66.2 ± 1.9 ^b^	71.1 ± 2.0 ^a^	57.8 ± 1.6 ^y^	61.8 ± 1.6 ^y^	72.7 ± 0.7 ^x^
Straightness (%)	82.4 ± 0.8	82.6 ± 0.7	83.6 ± 1.1	80.4 ± 1.1 ^y^	81.3 ± 0.7 ^y^	86.1 ± 0.3 ^x^
Wobble (%)	75.4 ± 2.1 ^c^	79.8 ± 1.8 ^b^	84.8 ± 1.4 ^a^	70.0 ± 1.9 ^z^	75.2 ± 1.5 ^y^	84.2 ± 0.5 ^x^
Amplitude of lateral head displacement (µm)	2.2 ± 0.3	2.1 ± 0.1	1.9 ± 0.1	2.3 ± 0.2	2.0 ± 0.0	1.8 ± 0.1
Beat cross frequency (Hz)	23.9 ± 0.4 ^a^	21.2 ± 0.7 ^b^	21.0 ± 0.6 ^b^	22.6 ± 0.8 ^x^	20.8 ± 0.4 ^y^	21.2 ± 0.4 ^y^
Normal morphology (%)	86.7 ± 1.8 ^b^	83.1 ± 2.6 ^b^	94.4 ± 0.9 ^a^	67.8 ± 2.0 ^y^	65.1 ± 2.8 ^y^	86.1 ± 2.9 ^x^
Head defects (%)	4.3 ± 0.9 ^a^	3.1 ± 0.7 ^a^	2.0 ± 0.2 ^b^	10.2 ± 2.4 ^x^	9.4 ± 2.6 ^x^	5.6 ± 1.6 ^y^
Midpiece/tail defects (%)	3.2 ± 1.0 ^b^	10.0 ± 3.0 ^a^	1.2 ± 0.3 ^c^	11.0 ± 1.9 ^y^	17.0 ± 2.4 ^x^	4.6 ± 2.1 ^z^
Proximal protoplasmic droplet (%)	4.0 ± 1.2 ^a^	2.5 ± 1.0 ^a^	2.3 ± 0.7 ^b^	6.2 ± 1.6 ^x^	6.2 ± 1.2 ^x^	2.7 ± 0.8 ^y^
Distal protoplasmic droplet (%)	1.7 ± 0.9 ^a^	1.3 ± 0.4 ^a^	0.1 ± 0.1 ^b^	4.7 ± 1.4 ^x^	2.2 ± 1.0 ^x^	0.6 ± 0.2 ^y^
Intact plasma membrane (%)	89.3 ± 1.3 ^a,b^	86.3 ± 1.1 ^b^	89.5 ± 1.2 ^a^	79.4 ± 3.7 ^x,y^	78.7 ± 4.1 ^y^	82.8 ± 3.2 ^x^
Intact acrosome (%)	92.6 ± 1.3	91.2 ± 1.0	89.7 ± 2.1	85.4 ± 3.1	87.2 ± 2.7	85.4 ± 4.0
High mitochondrial membrane potential (%)	81.6 ± 3.2 ^b^	81.6 ± 2.0 ^b^	90.4 ± 1.0 ^a^	74.6 ± 5.0 ^y^	69.0 ± 6.4 ^y^	80.0 ± 4.6 ^x^
DNA fragmentation index (%)	10.5 ± 3.1 ^a^	8.2 ± 0.8 ^a^	5.6 ± 0.8 ^b^	20.0 ± 4.4 ^x^	14.5 ± 2.9 ^x^	8.7 ± 1.2 ^y^
TBA-RS (ng/10^6^ sperm)	8.3 ± 2.5 ^a^	3.2 ± 0.3 ^b^	3.2 ± 0.5 ^b^	8.9 ± 2.0 ^x^	4.2 ± 0.9 ^y^	4.2 ± 0.6 ^y^

Within a row of the optimal quality column, different superscripts ^a,b,c^ indicate significant differences (*p* < 0.05). Within a row of the suboptimal quality column, different superscripts ^x,y,z^ indicate significant differences (*p* < 0.05).

**Table 3:** Post-thawed semen parameters (mean ± SEM) of optimal quality ejaculates (normal morphology ≥ 80%) and suboptimal quality ejaculates (normal morphology between 60 and 79%). Samples were cryopreserved after standard centrifugation (Standard) or single-layer centrifugation (SLC).

	Optimal Quality		Suboptimal Quality	
**Parameter**	**Standard**	**SLC**	**Standard**	**SLC**
Total motility (%)	59.2 ± 3.8	53.4 ± 5.2	36.2 ± 5.1	38.6 ± 4.2
Progressive motility (%)	47.1 ± 3.7	43.4 ± 4.7	26.1 ± 4.7	28.8 ± 3.9
Curvilinear velocity (µm/s)	146.2 ± 6.9 ^a^	139.4 ± 6.4 ^b^	132.1 ± 6.9 ^x^	121.9 ± 6.3 ^y^
Straight line velocity (µm/s)	73.8 ± 4.0	74.6 ± 3.3	65.2 ± 3.4	64.2 ± 4.0
Average path velocity (µm/s)	97.5 ± 4.1	96.1 ± 3.8	85.3 ± 3.7	82.6 ± 3.8
Linearity (%)	50.3 ± 2.1 ^b^	53.5 ± 2.3 ^a^	49.3 ± 1.3 ^y^	52.2 ± 1.4 ^x^
Straightness (%)	75.2 ± 2.1	77.3 ± 2.2	76.1 ± 0.8	76.9 ± 1.4
Wobble (%)	66.6 ± 1.3 ^b^	68.9 ± 1.3 ^a^	64.4 ± 1.2 ^y^	67.7 ± 0.9 ^x^
Amplitude of lateral head displacement (µm)	2.2 ± 0.1	2.0 ± 0.0	2.1 ± 0.1	2.0 ± 0.0
Beat cross frequency (Hz)	21.9 ± 0.8	21.9 ± 0.9	21.3 ± 0.3	21.9 ± 0.6
Normal morphology (%)	68.9 ± 2.7 ^b^	80.2 ± 2.4 ^a^	59.2 ± 3.4 ^y^	76.1 ± 5.0 ^x^
Head defects (%)	12.0 ± 2.7	13.9 ± 2.8	13.2 ± 2.6	14.9 ± 3.8
Midpiece/tail defects (%)	12.8 ± 3.0 ^a^	3.9 ± 0.8 ^b^	20.0 ± 4.2 ^x^	4.8 ± 2.1 ^y^
Proximal protoplasmic droplet (%)	4.0 ± 1.4 ^a^	1.8 ± 1.0 ^b^	5.2 ± 1.6 ^x^	1.9 ± 0.4 ^y^
Distal protoplasmic droplet (%)	2.2 ± 1.0 ^a^	0.3 ± 0.2 ^b^	1.9 ± 0.5 ^x^	0.8 ± 0.5 ^y^
Intact plasma membrane (%)	59.6 ± 2.9 ^b^	62.3 ± 3.6 ^a^	42.3 ± 4.8 ^y^	48.6 ± 3.4 ^x^
Intact acrosome (%)	68.3 ± 3.2	67.7 ± 3.8	55.0 ± 5.0	59.1 ± 2.8
High mitochondrial membrane potential (%)	27.8 ± 4.2 ^b^	37.3 ± 4.5 ^a^	25.9 ± 4.0	24.9 ± 4.1
DNA fragmentation index (%)	6.8 ± 0.8	6.6 ± 0.9	13.6 ± 3.1	7.8 ± 0.8
TBA-RS (ng/10^6^ sperm)	2.6 ± 0.4	2.8 ± 0.4	4.3 ± 0.8	4.2 ± 0.7

Within a row of the optimal quality column, different superscripts ^a,b^ indicate significant differences (*p* < 0.05). Within a row of the suboptimal quality column, different superscripts ^x,y^ indicate significant differences (*p* < 0.05).

## Data Availability

The data presented in this study are available on request from the corresponding author.
